# Supplementary material for: Assessing the effect of cardiovascular disease on work productivity and financial loss among school teachers in Peninsular Malaysia: a nested case-control study
Source: PeerJ. 2024 Feb 12;12:e16906. doi: 10.7717/peerj.16906 (PMC10868520; doi:10.7717/peerj.16906)
Supplement: Supplemental Information 2 [file peerj-12-16906-s002.docx]

**Appendix – Eight questions from the locally validated, short Malay version of** **World Health Organization-Health and Work Performance Questionnaire**

1. What is your current monthly salary (inclusive of allowances)?

*Berapakah jumlah pendapatan bulanan anda (termasuk elaun) sekarang?*

1. Has a doctor ever informed you that you had a new chronic disease for the past eight years? If yes, choose whichever is applicable (Bronchial asthma/ chronic obstructive pulmonary disease, diabetes mellitus, chronic kidney disease, migraine, others – to specify)

*Pernahkah doktor beritahu anda bahawa anda menghidapi penyakit kronik baru sepanjang jangka masa lapan tahun yang lepas? Jika ya, sila pilih yang berkaitan (Asma/ penyakit paru-paru obstruktif kronik, kencing manis, penyakit buah pinggang, migrain, lain-lain – sila nyatakan)*

1. Has a doctor ever informed you that you had a new acute disease for the past one month? If yes, choose whichever is applicable [Upper respiratory tract infection, acute gastroenteritis, motor vehicle accident, coronavirus disease (COVID-19), pregnancy, others – to specify]

*Pernahkah doktor beritahu anda bahawa anda menghidapi penyakit akut baru sepanjang jangka masa satu bulan yang lepas? Jika ya, sila pilih yang berkaitan [Jangkitan saluran pernafasan atas, selesema perut,, kemalangan jalan raya,, penyakit koronavirus 2019 (COVID-19), kehamilan, lain-lain – sila nyatakan]*

1. How many hours does your employer expect you to work in a typical 7-day week?

*Berapakah jumlah jam yang diminta oleh majikan anda untuk bekerja dalam tempoh 7 hari seminggu yang biasa?*

1. In the past 4 weeks (28 days), how many days did you

*Sepanjang 4 minggu (28 hari) yang lalu, berapa harikah anda habiskan dengan*

- 1. Miss an entire work day because of problems with cardiovascular disease (CVD)?

*Tidak masuk bekerja sepanjang hari kerana masalah berkaitan dengan penyakit kardiovaskular?*

- 1. Miss an entire work day for any other reason (including vacation)?

*Tidak masuk bekerja sepanjang hari kerana sebab lain (termasuk bercuti)?*

- 1. Miss part of a work day because of problems with CVD?

*Tidak masuk bekerja sebahagian hari kerana masalah berkaitan dengan penyakit kardiovaskular?*

- 1. Miss part of a work day for any other reason (including vacation)?

*Tidak masuk bekerja sebahagian hari kerana sebab lain (termasuk bercuti)?*

1. About how many hours altogether did you work in the past 4 weeks (28 days)?

*Berapakah jumlah jam anda bekerja sepanjang 4 minggu yang lalu (28 hari)?*

1. On a scale from 0 to 10 where 0 is the worst job performance anyone could have at your job and 10 is the performance of a top worker, how would you rate the usual performance of most workers in a job similar to yours?

*Dengan menggunakan skala 0 hingga 10 di mana 0 adalah prestasi kerja terburuk dan 10 adalah prestasi kerja terbaik, bagaimanakah anda menilai prestasi kerja kebanyakan pekerja lain yang melakukan pekerjaan yang sama atau hampir sama dengan anda?*

1. Using the same 0 to 10 scale, how would you rate your overall job performance on the days you worked during the past 4 weeks (28 days)?

*Dengan menggunakan skala 0 hingga 10 yang sama, bagaimanakah anda menilai prestasi kerja anda sendiri pada hari anda bekerja sepanjang 4 minggu yang lalu (28 hari)?*
